# The Effects of Armed Conflict on Healthcare Delivery and Nutritional Services in Somalia: A Narrative Review

**DOI:** 10.1002/puh2.70335

**Published:** 2026-08-05

**Authors:** Abdullahi Hassan Elmi

**Affiliations:** ^1^ Department of Nursing and Midwifery, Dr. Sumait Hospital, Faculty of Medicine and Health Sciences SIMAD University Mogadishu Somalia

**Keywords:** armed conflict, conflict‐sensitive strategies, food security, health workforce, healthcare delivery, malnutrition, nutrition services, Somalia

## Abstract

Armed conflict remains one of the most significant barriers to health system stability and food security in fragile states, such as Somalia. This narrative review synthesizes literature published between 2016 and 2025 to examine how ongoing conflict has shaped healthcare delivery and nutrition services, identifying key challenges, and exploring potential mitigation strategies. A comprehensive search of PubMed, Scopus, Web of Science, and Google Scholar, supplemented by credible grey literature, revealed consistent patterns: The destruction of health infrastructure, loss of skilled personnel, disrupted supply chains, and widespread displacement have severely restricted access to essential care. Nutritional programs have been equally affected, with conflict‐induced food insecurity and inflation driving alarming rates of acute and chronic malnutrition, particularly among children, pregnant women, and lactating mothers. Evidence from cash‐based interventions, integrated outreach models, and conflict‐sensitive service planning highlights opportunities to improve health and nutrition outcomes despite insecurity, though scalability remains constrained by funding gaps and access limitations. Health and nutrition workers face high personal risk, low morale, and resource shortages, whereas system‐level weaknesses hinder sustained improvement. Addressing these challenges requires coordinated action to protect facilities and staff, secure food systems, expand adaptable service delivery, and strengthen surveillance for timely response. The findings reinforce that sustainable progress in Somalia's health and nutrition sectors depends on embedding resilience into both emergency and long‐term interventions.

## 1 Introduction

Armed conflict profoundly undermines healthcare delivery and nutritional services globally, particularly in fragile settings like Somalia, where prolonged instability has severely impaired the health system's capacity to meet population needs. Over the past three decades, ongoing armed conflicts have destroyed health infrastructure, disrupted essential services, and hindered the delivery of lifesaving interventions, leaving millions vulnerable to disease and malnutrition [1, 2]. This narrative review synthesizes existing literature to critically analyze how armed conflict has affected healthcare and nutrition services in Somalia, drawing on empirical studies, systematic reviews, and grey literature from 2016 to 2025.

The healthcare system in Somalia faces multifaceted challenges due to conflict‐related destruction and governance vacuums, which have weakened public health programs, reduced access to vaccines and medications, and limited health workforce availability [1]. The country's fragile surveillance mechanisms further impair early detection and response to health emergencies, including outbreaks of vaccine‐preventable diseases and malnutrition crises [3]. Somalia's universal health coverage (UHC) index remains alarmingly low at 33.5, reflecting profound inequities in access and quality of healthcare services exacerbated by persistent insecurity and poverty [4].

Nutritional outcomes in Somalia mirror the deterioration of healthcare access, with high prevalence of acute and chronic malnutrition driven by conflict‐induced food insecurity, displacement, and disrupted caregiving practices [2, 5]. Conflict exacerbates vulnerabilities by limiting the delivery of nutrition‐specific interventions such as supplementation and therapeutic feeding programs [5]. Quantitative analyses reveal a positive correlation between armed conflict intensity, food price inflation, and increased rates of global acute malnutrition among children under five [6]. Climate variability compounds these effects, further stressing already fragile food systems [7].

Systematic reviews across conflict‐affected African regions emphasize similar trends, identifying armed conflict as a key determinant of childhood undernutrition. Factors, such as maternal education level, rural residence, and prolonged conflict duration, significantly influence malnutrition prevalence, underscoring the complex socio‐economic dimensions of the crisis [8]. These findings highlight the urgent need for context‐specific, resilient nutrition programs capable of operating in unstable environments.

Innovative health delivery models have emerged to address access gaps in conflict zones. The “Health Camp” mobile outreach program, implemented from 2022 to 2024, targeted hard‐to‐reach populations in areas controlled by non‐state armed actors. This initiative integrated primary healthcare with nutrition services, achieving a 56% reduction in zero‐dose children and vaccinating over 67,000 children across 396 camp sessions while screening nearly 100,000 children for malnutrition [1, 9]. Such models demonstrate the potential for adaptable service delivery that circumvents traditional barriers posed by insecurity. Despite these advances, significant challenges remain in scaling up health and nutrition services equitably across Somalia. Continued conflict, infrastructural deficits, and fragmented governance impede sustainable improvements, demanding coordinated efforts from government bodies, international agencies, and local communities [1, 2]. This review aims to consolidate current evidence on the effects of armed conflict on healthcare delivery and nutrition in Somalia, thereby informing strategic interventions to strengthen resilience and promote equitable access to essential services.

This review is guided by a conflict‐sensitive health systems and resilience framework, which recognizes that armed conflict affects healthcare delivery and nutritional outcomes through multiple interconnected pathways. These pathways include insecurity, forced displacement, destruction of health infrastructure, depletion of the health workforce, disruption of supply chains, weakened disease surveillance, and instability in livelihoods and food systems. Within this framework, healthcare delivery and nutrition are treated as closely linked but analytically distinct domains, both shaped by the broader political, economic, and humanitarian consequences of protracted conflict. This perspective provides a clearer structure for interpreting the evidence and for identifying response strategies that support both immediate service continuity and longer term system resilience.

## 2 Methods

### 2.1 Literature Search Strategy

This narrative review aimed to consolidate and critically analyze the existing literature on how armed conflicts have affected healthcare and nutrition services in Somalia. A systematic and comprehensive search was performed across major electronic databases, PubMed, Scopus, Web of Science, and Google Scholar, covering publications from May 2016 to May 2025 to ensure the inclusion of the most recent evidence. The search strategy incorporated carefully selected keywords combined with logical operators, using the following structure: “armed conflict” AND “Somalia” AND (“health services” OR “nutrition services” OR “healthcare system” OR “nutrition programs”).

### 2.2 Inclusion and Exclusion Criteria

The review focused on sourcing high‐quality evidence, giving priority to peer‐reviewed journal articles, systematic reviews, meta‐analyses, and scholarly commentaries. In addition, gray literature, including reports from government agencies, United Nations bodies, reputable nongovernmental organizations, and civil society groups, was considered. Eligible studies were those presenting empirical data and relevant contextual insights on healthcare and nutrition services in conflict‐affected areas of Somalia. Only English‐language publications were reviewed. Exclusion criteria encompassed duplicate records, studies unrelated to the topic, and articles without accessible full texts. This rigorous selection process was designed to reduce bias and facilitate a comprehensive, balanced synthesis of the available evidence.

## 3 Consequences of Armed Conflicts on Health and Nutrition Services

### 3.1 Impact of Armed Conflict on Healthcare Infrastructure and Access

The first pathway through which conflict affects population health in Somalia is the direct disruption of healthcare infrastructure, workforce capacity, and physical access to services. Armed conflict in Somalia has severely damaged healthcare infrastructure, drastically limiting the capacity to deliver essential health services. Prolonged violence and instability have led to widespread destruction or abandonment of hospitals and clinics, especially in rural and conflict‐prone areas. Attacks on health facilities and looting have further worsened the situation, reducing the number of operational service points and overwhelming those still functional [10]. This disruption contributes directly to diminished healthcare quality and availability. The healthcare workforce has also been critically affected. Many skilled health professionals have fled due to insecurity and threats to their lives, leading to acute shortages of qualified doctors, nurses, and technicians. This brain drain has further weakened the capacity of Somalia's already fragile health system [11]. Studies indicate that regions with ongoing conflict experience the most severe shortages, impairing critical services such as maternal health and emergency care [12].

Conflict‐induced displacement significantly restricts healthcare access. With over 2 million internally displaced persons (IDPs), many live in overcrowded camps with inadequate sanitation, clean water, and nutrition, exacerbating their health risks [13]. Insecurity and damaged infrastructure impede physical access to health facilities, delaying or preventing timely medical care [14]. Supply chains for essential medicines and vaccines are frequently disrupted by ongoing conflict and logistical challenges. Such interruptions result in stockouts that affect routine immunization and treatment of acute and chronic conditions [15]. Financial constraints, including low government health spending and fluctuating donor funding, further limit healthcare delivery and workforce retention [16].

These disruptions extend beyond clinical care alone. As conflict weakens service delivery, displaces communities, and undermines livelihoods, it also intensifies food insecurity and reduces access to nutrition services, thereby linking health system breakdown with worsening nutritional outcomes. Somalia's protracted conflict continues to degrade healthcare infrastructure and restrict access, underscoring the urgent need for protective measures for health facilities, investment in the health workforce, and innovative service delivery approaches to reach displaced and underserved populations. The evidence consistently shows that armed conflict in Somalia has led to widespread facility destruction, workforce depletion, and reduced access for displaced and rural populations. Disruptions to supply chains further weaken essential service delivery, particularly immunization and emergency care. These combined effects reveal a fragile health system unable to absorb ongoing shocks. Strengthening infrastructure protection and workforce retention remains central to restoring service continuity. These findings suggest that the effect of conflict on healthcare access is not only limited to physical destruction alone but also reflects the interaction of insecurity, displacement, supply instability, and weak financing, all of which reduce the system's ability to maintain routine and emergency care.

### 3.2 Effects on Nutritional Programs and Food Security

A second pathway operates through the disruption of food systems, livelihoods, and nutrition‐specific services, all of which intensify the risk of undernutrition in conflict‐affected communities. Armed conflict in Somalia has taken a heavy toll on nutrition programs and food security, with the greatest burden falling on children, pregnant women, and lactating mothers. Violence destroys essential infrastructure, displaces families, disrupts livelihoods, and weakens local markets, all of which make it harder for households to access sufficient and nutritious food [17, 18]. These disruptions are often worsened by food price inflation, which reduces purchasing power and further limits access to diverse diets, especially among poor and displaced communities. In Somalia, the nutritional effects of conflict are driven not only by interruptions in health and nutrition services, but also by severe disruption of livelihoods and food markets.

Armed conflict can displace farming and pastoral households, restrict trade routes, reduce market activity, and interrupt income‐generating opportunities, leaving many families with less money to buy food. These pressures are often compounded by food price inflation, which further reduces household purchasing power and makes nutritious diets increasingly unaffordable for low‐income communities [6]. As a result, families may shift to cheaper, less diverse foods, reduce meal frequency, or delay seeking care for nutrition‐related problems. These effects fall most heavily on children, pregnant women, and lactating mothers, whose nutritional needs are greater and whose health risks rise quickly when dietary intake deteriorates. In this way, conflict contributes to malnutrition through both direct service disruption and indirect economic and food‐system pathways.

Recent research in Somalia highlights how targeted interventions can help soften these blows. Cash‐based assistance, such as unconditional cash transfers or vouchers, has shown promise in improving nutrition outcomes amid ongoing crises. For instance, Doocy et al. found that both cash and voucher programs enhanced the nutrition status of pregnant and lactating women by increasing their ability to buy diverse and nutritious foods during the height of Somalia's food crisis [19]. Another study from the same group demonstrated similar positive impacts on children's nutrition, emphasizing that flexible cash support can be a vital lifeline in unstable settings [20].

Beyond cash aid, combining nutrition education with financial support amplifies benefits. Ali et al. [21] conducted a study in IDP camps, showing that nutrition counseling paired with cash transfers led to measurable improvements in child growth and family food security. This finding underscores the importance of empowering families with knowledge alongside resources to make better nutritional choices, even in difficult circumstances [21]. Taken together, these studies demonstrate that cash‐based interventions, especially when combined with nutrition counseling, consistently improve household food access and child nutrition outcomes in conflict‐affected settings. Across the literature, a clear pattern emerges showing that flexible financial support enables families to maintain dietary diversity even when markets and food systems are disrupted by conflict. Despite these encouraging results, challenges remain. Persistent insecurity, limited humanitarian access, and unpredictable funding mean that many at‐risk communities still struggle to get the support they need. Strengthening and scaling nutrition programs that blend cash assistance with education and community engagement will be essential to improve food security and health in Somalia's fragile environment.

Overall, conflict‐driven displacement, food insecurity, and market disruptions continue to fuel malnutrition across Somalia. Although cash transfers and nutrition counseling show measurable benefits, their reach is limited by insecurity and inconsistent funding. The literature highlights the importance of combining resources with education to improve outcomes. Continued investment in flexible, scalable nutrition programs is essential in high‐risk regions. This pattern indicates that nutritional deterioration in Somalia is driven by both service disruption and broader household vulnerability, reinforcing the need for responses that address immediate nutritional needs alongside the wider economic and food‐system effects of conflict.

### 3.3 Challenges Faced by Health and Nutrition Workforce in Conflict Zones

A third pathway involves pressure on frontline health and nutrition workers, whose safety, motivation, and ability to deliver care are undermined by insecurity and weak system support. Health and nutrition workers in conflict zones like Somalia face extraordinary challenges that strain their ability to provide vital care. Constant insecurity and violence create a dangerous working environment where attacks on health facilities and staff are all too common. Many health workers are forced to abandon their posts out of fear for their safety, leaving communities with critical gaps in care, especially in the hardest‐to‐reach and most volatile areas [22, 23]. This insecurity, combined with ongoing displacement, disrupts the healthcare workforce and breaks the continuity of essential services.

Beyond physical risks, motivation is a serious concern. A study in Mogadishu found that health workers often struggle with low morale due to irregular pay, lack of professional development opportunities, and limited psychosocial support. These factors make it difficult to retain skilled staff and sustain quality services under such stressful conditions [4, 23]. Moreover, many workers lack adequate training and supervision, which hampers their ability to manage complex nutrition and health challenges effectively.

System‐level barriers add another layer of difficulty. For example, bottlenecks in supply chains and poor coordination hinder the integrated management of acute malnutrition, leaving nutrition workers without the necessary tools and resources to carry out their work [24]. Collectively, the evidence illustrates how insecurity, poor working conditions, and weak system‐level support interact to undermine the capacity and motivation of health and nutrition workers. These interconnected challenges create a cycle in which staff shortages, limited training, and resource gaps reinforce each other, ultimately reducing the quality and continuity of care in conflict zones. Compounding these issues, displaced populations face overlapping vulnerabilities like poverty and gender‐based violence, which require sensitive, multifaceted responses from health teams [7, 25]. To overcome these hurdles, protecting health workers and improving their working conditions is critical. Strengthening health systems to withstand conflict shocks, ensuring ongoing training, and offering psychological support can help sustain the workforce. Ultimately, investing in the people who deliver care is essential to maintaining vital health and nutrition services in Somalia's unstable environment. Health and nutrition workers face interconnected challenges, including insecurity, low motivation, inadequate training, and persistent supply shortages. These pressures compromise service quality and disrupt continuity of care in high‐need communities. Evidence emphasizes the need for stronger workforce protection, improved supervision, and psychosocial support. Sustaining frontline staff is a prerequisite for effective health and nutrition delivery in conflict zones. Together, these findings show that workforce challenges are not isolated operational problems, but central system‐level constraints that directly shape the continuity, quality, and reach of health and nutrition services during conflict.

### 3.4 Strategies to Mitigate the Health and Nutrition Consequences of Armed Conflicts

In response to these overlapping pathways, mitigation strategies must address both immediate humanitarian needs and longer term system resilience. Mitigating the health and nutrition consequences of armed conflict requires more than emergency relief alone. In Somalia, effective response depends on addressing both the direct disruption of healthcare and nutrition services and the broader structural pressures that conflict places on displacement, livelihoods, food systems, and access to vulnerable populations. For this reason, mitigation strategies must combine short‐term humanitarian action with longer term measures that strengthen resilience, continuity of care, and local system capacity.

**Emergency health and nutrition interventions** remain essential during periods of active conflict because fixed facilities are often destroyed, inaccessible, or unable to function consistently. In such settings, mobile health clinics and community health workers help preserve continuity of care by bringing essential services closer to displaced and hard‐to‐reach populations [26, 27]. These models are especially important for maternal and child health, vaccination, and the early identification and treatment of acute malnutrition. Their value lies not only in temporary service substitution, but also in their flexibility, which allows delivery platforms to adapt as insecurity, displacement patterns, and access constraints change over time [26].

Resilience‐focused strategies are equally important because repeated shocks can quickly reverse gains made through short‐term humanitarian action. Integrating conflict sensitivity into health system planning means designing services that can continue operating despite insecurity, population movement, and disruptions in supplies or staffing [28, 29]. In practice, this may include decentralized service delivery, local workforce support, contingency planning, and stronger coordination between humanitarian and public health actors. Evidence from Somalia suggests that when long‐term monitoring is paired with flexible delivery models, health and nutrition services can remain more responsive even in highly unstable settings, including IDP camps [30].

Food security measures must extend beyond short‐term aid because emergency assistance alone cannot address the structural drivers of nutritional deterioration in conflict‐affected settings. In Somalia, protracted armed conflict undermines food security through multiple overlapping mechanisms, including displacement, disrupted agricultural production, restricted market access, damaged trade networks, loss of livelihoods, and weak governance [17, 31]. These factors not only reduce food availability but also erode household purchasing power, especially when combined with insecurity and inflation. Consequently, food insecurity should be understood as a persistent consequence of conflict rather than a short‐lived emergency [28, 32]. A more effective long‐term response therefore requires protected humanitarian access, support for local food production, livelihood recovery, market stabilization, and stronger governance structures. At the same time, meaningful and sustained progress will remain difficult without broader peacebuilding and stability‐oriented efforts that address the root drivers of repeated crisis [28, 32, 33].

Alongside service delivery and food security interventions, effective mitigation also depends on strong systems for tracking evolving needs and guiding timely response. Data and surveillance systems are an essential component of conflict‐sensitive health and nutrition response. In Somalia, protracted insecurity can disrupt routine health information systems by restricting access, fragmenting reporting pathways, displacing communities, and weakening local institutional capacity. These disruptions reduce the visibility of emerging outbreaks, mortality patterns, and malnutrition trends, thereby delaying timely response and weakening evidence‐based decision‐making. In contexts where civil registration and formal cause‐of‐death systems are absent or incomplete, alternative methods such as verbal autopsy can generate critical information on mortality and morbidity, especially in IDP camps and other insecure settings [30, 34]. Framed in this way, surveillance is not a separate technical issue, but a core part of effective planning, targeting, and resilience‐building in health and nutrition services.

Taken together, the evidence suggests that effective mitigation in Somalia depends on combining flexible frontline delivery with longer term system strengthening, food security support, and better data for decision‐making. A fragmented response is unlikely to be sufficient; rather, progress depends on coordinated strategies that address both immediate humanitarian needs and the deeper structural effects of protracted conflict.

## 4 Limitations of Current Review

This review is subject to several limitations. First, the reliance on published literature and accessible grey sources may have excluded relevant field data or operational reports not publicly available, potentially limiting the comprehensiveness of the evidence base. Second, variability in study design, sample sizes, and methodological rigor among included sources makes direct comparison of findings challenging and may introduce bias. Third, although the review integrates both national and regional perspectives, it may underrepresent local community‐level insights, particularly from inaccessible conflict zones where data collection remains constrained.

## 5 Future Research Directions

Future research should focus on generating stronger, locally grounded evidence from remote and conflict‐affected communities, particularly IDP camps where data gaps are common. Studies using mixed‐methods and longitudinal designs are needed to capture both trends and lived experiences over time. Integrating real‐time data tools, such as mobile health reporting and geospatial monitoring, could improve tracking of how conflict disrupts health and nutrition services.

There is also a need to evaluate the effectiveness, cost‐efficiency, and scalability of current interventions, including cash assistance, nutrition counseling, and mobile outreach models, especially in insecure areas. Research should further explore conflict‐sensitive service delivery approaches and how they can be sustained during active violence. Finally, future work must give more attention to underserved groups, such as adolescents, people with disabilities, minority clans, and isolated rural communities. Strengthening evidence in these areas will support more inclusive policies and more resilient health and nutrition systems in Somalia.

## 6 Conclusion

This review underscores the profound and multifaceted impacts of armed conflict on healthcare delivery and nutrition services in Somalia. Destruction of infrastructure, loss of skilled health workers, disrupted supply chains, and restricted access to vulnerable populations have combined to create a protracted humanitarian and public health crisis. Malnutrition rates remain unacceptably high, driven by conflict‐induced food insecurity and displacement.

Although innovative responses, such as mobile health camps, integrated cash and nutrition programs, and conflict‐sensitive service delivery, have shown measurable benefits, their reach remains limited due to insecurity, fragmented governance, and unstable funding. Strengthening health systems in Somalia demands coordinated action between government, humanitarian actors, and local communities. Priorities should include safeguarding health facilities, investing in workforce protection and motivation, supporting sustainable food production, and enhancing surveillance systems to ensure timely, data‐driven interventions.

Addressing these issues is not only a humanitarian imperative but also central to rebuilding resilience, equity, and stability in Somalia's health and nutrition sectors.

## Author Contributions

Abdullahi Hassan Elmi was solely responsible for the conception and design of the study, data collection, analysis, interpretation, and drafting of the manuscript. The author approves the final version and agrees to be accountable for all aspects of the work.

## Funding

This research Supported by SIMAD University

## Ethics Statement

The author has nothing to report.

## Consent

The author has nothing to report.

## Conflicts of Interest

The author declares no conflicts of interest.

## Data Availability

The data that support the findings of this study are available from the corresponding author upon reasonable request.

## References

[1] A. Y. Mohamed , A. H. Hassan , and M. A. Abdullahi , The Challenges Facing the Healthcare System in Somalia: A Review and the Way Forward (2023), https://www.researchgate.net/publication/378108504_The_challenges_facing_the_healthcare_system_in_Somalia_A_review_and_The_Way_Forward.

[2] United Nations Children's Fund (UNICEF) , Impact of Armed Conflict on Child Nutrition and Health in Somalia , UNICEF Somalia Reports (UNICEF, 2023).

[3] R. Martin‐Canavate , E. Custodio , A. Yusuf , D. Molla , D. Fasbender , and F. Kayitakire , “Malnutrition and Morbidity Trends in Somalia Between 2007 and 2016: Results From 291 Cross‐Sectional Surveys,” BMJ open 10, no. 2 (2020): e033148, 10.1136/bmjopen-2019-033148. PMC7045078 32071180

[4] World Health Organization , Somalia Healthcare Coverage Index Report (WHO Global Health Observatory, 2024), https://pubmed.ncbi.nlm.nih.gov/40336100/.

[5] World Food Programme (WFP) , Food Security Challenges in Somalia Due to Conflict and Climate Change , WFP Somalia Annual Report (WFP, 2023).

[6] J. Mohamed , M. J. Abdi , A. I. Mohamed , et al., “Predicting the Short and Long Term Effects of Food Price Inflation, Armed Conflicts, and Climate Variability on Global Acute Malnutrition in Somalia,” Journal of Health, Population and Nutrition 43 (2024): 68, 10.1186/s41043-024-00557-9. 38760867 PMC11102243

[7] A. H. Hassan and A. H. Elmi , “Healthcare Delivery Innovations in Conflict Zones: The Somali Experience,” Conflict and Health Journal 16, no. 1 (2022): 29–38.

[8] M. M. Azanaw , D. T. Anley , R. M. Anteneh , G. Arage , and A. A. Muche , “Effects of Armed Conflicts on Childhood Undernutrition in Africa: A Systematic Review and Meta‐Analysis,” Systematic Reviews 12 (2023): 46, 10.1186/s13643-023-02206-4. 36922839 PMC10015806

[9] Save the Children , Health Camp: An Alternate Model to Deliver Health and Nutrition Services Ensuring Equity and Accessibility for Areas Controlled by Non‐State Armed Actors in Somalia (Save the Children's Resource Centre, 2024), https://resourcecentre.savethechildren.net/document/health‐camp‐an‐alternate‐model‐to‐deliver‐health‐and‐nutrition‐services‐ensuring‐equity‐and‐accessibility‐for‐areas‐controlled‐by‐non‐state‐armed‐actors‐in‐somalia/.

[10] C. Robinson , N. Spicer , and E. Wainwright , “The Impact of Conflict on Healthcare Systems in Somalia: A Scoping Review,” Conflict and Health 15, no. 1 (2021): 1–15.33390172

[11] A. Warsame , H. Ismail , and M. Barre , “Health and Nutrition Challenges Among Internally Displaced Persons in Somalia: A Literature Review,” Journal of Refugee Studies 33, no. 4 (2020): 675–692.

[12] M. Hassan and S. A. Ali , “Barriers to Healthcare Access Among Displaced Populations in Somalia: A Mixed‐Methods Study,” BMC Public Health [Electronic Resource] 22, no. 1 (2022): 1057.35619059

[13] UNHCR Somalia , Somalia Situation: Health and Nutrition Update (UNHCR, 2023), https://data.unhcr.org/en/documents/details/10421.

[14] Oxfam International , Barriers to Healthcare Access in Somalia's IDP Camps (Oxfam International, 2023), https://policy‐practice.oxfam.org/resources/barriers‐to‐healthcare‐access‐in‐somalia‐620007.

[15] M. Ali and F. Osman , “Impact of Conflict on Vaccine Supply Chains in Somalia,” Vaccine 40, no. 15 (2022): 2267–2274.

[16] Global Fund , Financing Health Services in Fragile Settings: Somalia Case Study (Global Fund, 2023), https://www.theglobalfund.org/en/blog/2023‐01‐23‐financing‐health‐services‐in‐fragile‐settings‐somalia‐case‐study/.

[17] A. Kadir , S. Shenoda , and J. Goldhagen , “Effects of Armed Conflict on Child Health and Development: A Systematic Review,” PLoS ONE 14, no. 1 (2019): e0210071, 10.1371/journal.pone.0210071. 30650095 PMC6334973

[18] S. Garry and F. Checchi , “Armed Conflict and Public Health: Into the 21st Century,” Journal of Public Health 42, no. 3 (2020): e287–e298, 10.1093/pubmed/fdz095. 31822891

[19] S. Doocy , M. Busingye , E. Lyles , et al., “Cash‐Based Assistance and the Nutrition Status of Pregnant and Lactating Women in the Somalia Food Crisis: A Comparison of Two Transfer Modalities,” PLoS ONE 15, no. 4 (2020): e0230989, 10.1371/journal.pone.0230989. 32324761 PMC7179869

[20] S. Doocy , M. Busingye , E. Lyles , et al., “Cash and Voucher Assistance and Children's Nutrition Status in Somalia,” Maternal & Child Nutrition 16, no. 3 (2020): e12966, 10.1111/mcn.12966. 32141183 PMC7296788

[21] M. K. Ali , R. Flacking , M. Sulaiman , and F. Osman , “Effects of Nutrition Counselling and Unconditional Cash Transfer on Child Growth and Family Food Security in Internally Displaced Person Camps in Somalia—A Quasi‐Experimental Study,” International Journal of Environmental Research and Public Health 19, no. 20 (2022): 13441, 10.3390/ijerph192013441. 36294019 PMC9603782

[22] M. F. Gaffey , A. Ataullahjan , J. K. Das , et al., “Researching the Delivery of Health and Nutrition Interventions for Women and Children in the Context of Armed Conflict: Lessons on Research Challenges and Strategies From BRANCH Consortium Case Studies of Somalia, Mali, Pakistan and Afghanistan,” Conflict and Health 14, no. 1 (2020): 69, 10.1186/s13031-020-00320-4. 33088339 PMC7574460

[23] N. S. Sheikh and A. A. Gele , “Factors Influencing the Motivation of Maternal Health Workers in Conflict Setting of Mogadishu, Somalia,” PLOS Global Public Health 3, no. 3 (2023): e0001673, 10.1371/journal.pgph.0001673. 36963062 PMC10021578

[24] J. Ntambi , M. A. Abdirahman , D. Nabiwemba , et al., “Bottleneck Analysis for the Integrated Management of Acute Malnutrition Services in Somalia,” Field Exchange 60 (2019): 56–60.

[25] J. Kelly , M. Holmes , N. Gibbons , A. Matabaro Tom , and M. Voors , “Conflict, Displacement and Overlapping Vulnerabilities: Understanding Risk Factors for Gender Based Violence Among Displaced Women in Eastern Democratic Republic of Congo,” Journal of Development Studies 60, no. 12 (2024): 2022–2049, 10.1080/00220388.2024.2376398.

[26] R. Dahab , L. Bécares , and M. Brown , “Armed Conflict as a Determinant of Children Malnourishment: A Cross‐Sectional Study in the Sudan,” BMC Public Health [Electronic Resource] 20, no. 1 (2020): 532, 10.1186/s12889-020-08665-x. 32306937 PMC7168991

[27] M. W. Arage , H. Kumsa , M. S. Asfaw , et al., “Exploring the Health Consequences of Armed Conflict: The Perspective of Northeast Ethiopia, 2022: A Qualitative Study,” BMC Public Health [Electronic Resource] 23, no. 1 (2023): 2078, 10.1186/s12889-023-16983-z. 37875885 PMC10594710

[28] E. Bendavid , T. Boerma , N. Akseer , et al., “The Effects of Armed Conflict on the Health of Women and Children,” Lancet 397, no. 10273 (2021): 522–532, 10.1016/S0140-6736(21)00131-8. 33503456 PMC7612212

[29] A. A. Warsame , A. M. Abdullahi , and H. A. Hussein , “Modeling the Impact of Conflicts and Socioeconomic Factors on Mortality Rate in Somalia,” Discover Sustainability 6, no. 1 (2025): 439, 10.1007/s43621-025-01043-w.

[30] A. J. Seal , M. Jelle , C. S. Grijalva‐Eternod , H. Mohamed , R. Ali , and E. Fottrell , “Use of Verbal Autopsy for Establishing Causes of Child Mortality in Camps for Internally Displaced People in Mogadishu, Somalia: A Population‐Based, Prospective, Cohort Study,” Lancet Global Health 9, no. 9 (2021): e1286–e1295, 10.1016/S2214-109X(21)00254-0. 34416214

[31] M. Hussein , C. Law , and I. Fraser , “An Analysis of Food Demand in a Fragile and Insecure Country: Somalia as a Case Study,” Food Policy 101 (2021): 102092, 10.1016/j.foodpol.2021.102092.

[32] J. A. Tyndall , K. Ndiaye , C. Weli , et al., “The Relationship Between Armed Conflict and Reproductive, Maternal, Newborn and Child Health and Nutrition Status and Services in Northeastern Nigeria: A Mixed‐Methods Case Study,” Conflict and Health 14, no. 1 (2020): 75, 10.1186/s13031-020-00318-5. 33292426 PMC7664044

[33] C. Oberg , H. Hodges , and A. S. Masten , “Risk and Resilience of Somali Children in the Context of Climate Change, Famine, and Conflict,” Journal of Applied Research on Children 12, no. 1 (2021): 10.

[34] R. Martin‐Canavate , E. Custodio , A. Yusuf , D. Molla , D. Fasbender , and F. Kayitakire , “Malnutrition and Morbidity Trends in Somalia Between 2007 and 2016: Results From 291 Cross‐Sectional Surveys,” BMJ Open 10, no. 2 (2020): e033148, 10.1136/bmjopen-2019-033148. PMC7045078 32071180

